# NCAPG promotes the proliferation of hepatocellular carcinoma through the CKII-dependent regulation of PTEN

**DOI:** 10.1186/s12967-022-03519-z

**Published:** 2022-07-21

**Authors:** Rongguiyi Zhang, Jiyuan Ai, Jiakun Wang, Chi sun, Hongcheng Lu, Aoxiao He, Min Li, Yuting Liao, Jun Lei, Fan Zhou, Linquan Wu, Wenjun Liao

**Affiliations:** 1 Department of General Surgery, The Second Affiliated Hospital of Nanchang University, No. 1, Minde Road, Nanchang, 330006 China; 2 Department of General Surgery, The Third Hospital of Nanchang City, No. 2, Xiangshan South Road, Nanchang, 330006 China; 3 Department of Nursing, Gannan Medical College, No. 1, Medical Road, Ganzhou, 341000 China

**Keywords:** Hepatocellular carcinoma (HCC), Proliferation, Non-SMC Subunit in the Concentrate I Complex (NCAPG), Phosphatase and Tensin Homolog (PTEN), Casein Kinase 2 Alpha 1 (CKII)

## Abstract

**Background:**

NCAPG, non-SMC subunit in the concentrate I complex, might promote the proliferation of hepatocellular carcinoma (HCC), but the mechanism is unclear. The aim of this study was to explore how NCAPG affects PTEN to influence the proliferation of HCC.

**Methods:**

Western blotting, qRT-PCR and immunohistochemistry were used to detect NCAPG expression in HCC tissues. The effect of NCAPG on the proliferation of HCC cell lines was evaluated using an EdU incorporation assay, a Cell Counting Kit-8 assay and Fluorescence in situ hybridization (FISH). BALB/c-nu/nu mice were used for the in vivo proliferation experiment. Transcriptome sequencing was used to determine the relationship between NCAPG and PTEN. Immunocoprecipitation-mass spectrometry (IP-MS), proteomic sequencing and Co-immunoprecipitation (CO-IP) were used to identify and examine the interaction between the NCAPG and CKII proteins.

**Results:**

We confirmed that NCAPG was abnormally overexpressed in HCC and promoted the proliferation of HCC cells. Transcriptome sequencing revealed that NCAPG inhibited the transcription of PTEN and promoted the activation of the PI3K-AKT pathway. We found a close association between NCAPG and CKII through proteomic sequencing; their interaction was confirmed by Co-IP. There was a positive correlation between NCAPG and CKII that promoted the phosphorylation of PTEN and thus inhibited its transcription and functions. We also proved that CKII was the key factor in the induction of proliferation by NCAPG.

**Conclusion:**

We revealed the mechanism by which NCAPG regulates the proliferation of HCC: NCAPG inhibits PTEN through its interaction with CKII, and then activates the PI3K-AKT pathway to promote the proliferation of HCC.

## Introduction

Hepatocellular carcinoma (HCC) is currently the sixth most common malignancy worldwide and has the third highest mortality rate [1]. Because HCC is prone to exhibit early vascular invasion, capsular invasion and intrahepatic and extrahepatic metastasis, the current treatments are not ideal [2]. Hence, it is essential to explore the mechanism of tumorigenesis in HCC. Studies have shown that abnormal proliferation is an important part of the development of liver cancer [3].Therefore, actively exploring the molecular mechanisms that affect the proliferation of HCC can not only help to understand its occurrence and development, but also lead to the discovery of key targets. Such studies will facilitate advances in the clinical diagnosis and treatment of HCC and the development of drugs for this malignancy.

NCAPG is a non-SMC subunit in the concentrate I complex that can encode certain condensin protein complexes [4]. This gene was confirmed to affect mitosis and found to be abnormally expressed in some tumours, such as glioma, prostate cancer, and HCC in our previous studies [5, 6]. HK Man emphasized that whole-genome knockout screening has identified NCAPG as a clinical target necessary for the growth of HCC and that targeting this gene will provide a new approach for the diagnosis and treatment of HCC [7]. In our previous studies, we confirmed that NCAPG has abnormally high expression in liver cancer [8]. Here, we explored the mechanism by which NCAPG enhances the proliferation of HCC.

PTEN (phosphatase and tensin homolog) is considered to be a key negative regulator of the PI3K-AKT pathway [9]. PTEN can dephosphorylate the second messenger PI(3,4,5)P3 and generate PI(4,5)P2, which interferes with AKT phosphorylation and exerts an inhibitory effect on the pathway [10]. Chalhoub pointed out in a previous study that PTEN mutation or loss of function is closely related to the occurrence of many tumours [11]. Recent studies indicate that deletion of the PTEN gene may be one of the mechanisms of drug resistance in tumours and an important reason for the poor clinical efficacy of targeted drug therapy [12]. Leonardo Salmena also proposed that exploring the upstream genes that affect PTEN can help solve the problem of abnormal tumour proliferation [13]. Thus, the upstream genes that regulate PTEN may play an important role in the occurrence and development of tumors.

Casein Kinase 2 Alpha 1 (CKII) is a serine/threonine protein kinase with structural activity. Its main functions are to control substrate selectivity and enzyme stability [14, 15]. The latest research found that CKII can induce the phosphorylation of PTEN [16, 17]. Phosphorylation is an important posttranscriptional modification that plays an important role in the regulation of PTEN [18]. After PTEN is regulated by phosphorylation, the amino acid at its C-terminus is closed due to the addition of a phosphate group, which ultimately leads to functional inhibition and the inability to regulate the PI3K-AKT signaling pathway [19, 20]. In addition, phosphorylated PTEN has been proven to inhibit the transcription of PTEN though NF-κB activation [21]. Thus, phosphorylation of PTEN leads to inhibition of both the function and transcription of PTEN [22, 23].

In this study, we aimed to reveal the mechanism by which NCAPG enhances the proliferation of HCC. We believe that this regulatory mechanism might be a novel pathway by which NCAPG promotes HCC proliferation.

## Methods

### Tissue specimens

A total of 85 hepatocellular carcinoma tissue specimens were collected between 2019 and 2021 for this study. Each sample was taken after liver resection without treatment prior to surgery. The liver cancer tissues and adjacent tissues of the specimens were collected and immediately placed in liquid nitrogen for preservation or stored at − 80 ℃ for subsequent use. This study was approved by the Ethical Review Committee of the Second Affiliated Hospital of Nanchang University. The whole experimental process was carried out in strict accordance with the ethical standards of the committees responsible for human experiments (institutional and national) and the Helsinki Declaration from 1975. Written informed consent was obtained from each patient before enrollment.

### In vivo proliferation experiment

For the in vivo proliferation test, 1 × 10^7^ cells in 100 µL of PBS were injected subcutaneously into female BALB/c-nu/nu mice (6–8 weeks old; n = 5 per group) (Hunan SJA Laboratory Animal Co., Ltd., Hunan, China). After 6 weeks, the mice were sacrificed under anesthesia, the tumor mass was removed, and the volume of the tumor mass was measured. In addition, HE was used to verify that the mass was liver cancer, and immunohistochemistry was used to compare the differences in NCAPG expression and proliferation-related protein expression among groups. All animal experiments were approved by the Animal Experiment Ethics Committee of the Laboratory Animal Science Center of Nanchang University and were carried out in accordance with the guidelines of the British Animal (Scientific Procedure) Act of 1986 and the European Union Directive 2010/63/EU.

### Cell culture

In this study, two HCC cell lines (MHCC97H and HCCLM3) were used, both of which were purchased from Zhejiang Meisen Cell Technology Co., Ltd. (Pan'an, Zhejiang, China). Complete medium containing 10% FBS (Biological Industries, Beit-Haemek, Israel), 100 µg/mL streptomycin and 100 U/mL penicillin in high-sugar DMEM (Solarbio, Beijing, China) was prepared as a suitable medium for all cell lines in this study. The cells were cultured in a humidified incubator with 5% CO_2_ at 37 °C.

### Transcriptome sequencing

RNA was extracted from HCCLM3 cells either overexpressing NCAPG or with NCAPG knockdown for RNA-Seq analysis. Strict quality control was performed on these RNA samples as follows: analysis of RNA integrity and DNA contamination (by agarose gel electrophoresis), detection of RNA purity (with a NanoPhotometer ^®^ spectrophotometer) and accurate detection of RNA integrity (with an Agilent 2100 bioanalyzer). After quality control, we obtained mRNA from the RNA, randomly interrupted the obtained mRNA with divalent cations in NEB fragmentation buffer, and built a library according to the NEB normal library building method or the chain-specific library building method. After the library was constructed, a Qubit ^®^2.0 fluorometer (Life Technologies) was used for preliminary quantification. The library was diluted to 1.5 ng/ul and then an RNA Nano 6000 Assay Kit from the Bioanalyzer 2100 system (Agilent Technologies, CA, USA) was used to detect the insert size of the library. After the insert size met the expectations, qRT-PCR was performed to measure the effective concentration of the library. Accurate quantification (the effective concentration of the library was greater than 2 nM) was performed to ensure the quality of the library. After the library was qualified, the different libraries were pooled according to their effective concentrations and target off-machine data volume for Illumina ^®^ sequencing and subsequent data analysis.

### Proteomics analysis

Proteomics analysis generally consists of the following four steps: sample treatment, in-gel digestion, IP-MS analysis, and data analysis. First, the strips were cut into 0.5–1 mm^3^ cubes, and incubated with decolorizing solution (50% acetonitrile in 50 mM triethylammonium bicarbonate) until decolorization was complete. Acetonitrile was added for dehydration and the samples were allowed to air dry naturally. Second, the gel pieces were reswollen on ice following the dropwise addition of 10–20 µL of TEAB containing 10 ng/µL trypsin (Promega), and the proteins were digested overnight at 37 °C. After digestion, the residual liquid was collected, and the remaining peptides were extracted into 100 µL of 0.1% formic acid (FA). After combining the two portions, the peptides were desalted with a C18 cartridge and dried by vacuum centrifugation. Third, mobile phases A (water with 0.1% formic acid) and B (80% acetonitrile, 0.1% formic acid) were prepared. Then, 10 µL of mobile phase A was used to dissolve the lyophilized powder followed by centrifugation at 12,000 rpm at room temperature for 5 min. The supernatant (1 µg) was injected for liquid quality testing. A q-Exactive™ HFX mass spectrometer was used for primary mass spectrometry, and the top 40 parent ions from the full scan were selected for fragmented by the high-energy impact pyrolysis (HCD) method for secondary mass spectrometry measurements to generate the original mass spectrometry detection data. Finally, data analysis was performed.

### Fluorescence in situ hybridization (FISH)

Fluorescence in situ hybridization (FISH) is a cytogenetic technique. In this technology, fluorescence probes are combined with chromosomes by an in situ hybridization method that replaces isotope markers with fluorescence markers. Qualitative or quantitative analysis of target genes by binding site and number can be carried out with FISH. The wax blocks were dewaxed, incubated with protease K solution at 37 ℃ for 20 min and then dehydrated with a gradient alcohol series. Each slice was incubated at 78 ℃ for 8 min in denaturation solution. Then, the cells were immediately incubated at − 20 ℃ for precooling in a gradient of alcohol solutions for 2 min. Next, the slices were placed across from each other in a large wet box. Ten microliters of probe was dropped onto each section which was then covered with cover glass. The wet box was covered and the sections were incubated at 37 ℃ for 12–16 h. After hybridization, the sections were washed with a water solution preheated at 43 ℃ for 15 min. Subsequently, each slice was incubated with 30–60 µl of FITC ovalbumin at room temperature for 20 min (repeated three times). Finally, each slice was incubated with 10–20 µl of DAPI at room temperature for 2–5 min. The cells were observed under a fluorescence microscope as soon as possible after staining.

### Immunohistochemistry

Briefly, a paraffin-embedded sample was sliced on a glass slide into 4 µm sections. After the slices were deparaffinized, hydrated, and incubated with 0.3% hydrogen peroxide for 15 min to block endogenous peroxidase, they were put into sodium citrate buffer (10 mmol/L, pH 6.0) for microwave heating at 10 ℃ for 10 min for antigen retrieval. Next, goat serum was used for blocking. Then, the sections were placed into a prepared anti-NCAPG rabbit monoclonal antibody solution (1:200; 24,563-1-AP, Proteintech, Wuhan, Hubei, China) and incubated overnight at 4 ℃. The next day, the cells were washed with PBS 3 times for 5 min each time. Then, the sections were placed into a secondary biotinylated goat anti-mouse IgG solution and incubated at 37 ℃ for 30 min. The sections were stained with diaminobenzidine and hematoxylin and then sealed with neutral resin. The staining intensity and percent positive cells were scored blindly, randomly and semiquantitatively. The overall staining index was calculated by multiplying the grade and the score to reach a value between 0 and 9, which was finally designated as NCAPG non-overexpression (0–1) or NCAPG overexpression (2–9).

### Transfection

The NCAPG overexpression plasmid (pcDNA3.1( +)-NCAPG) and corresponding negative control vector (pcDNA3.1( +)-NC), NCAPG knockdown plasmid (shNCAPG) and control (shNC), and CKII overexpression (pcDNA3.1( +)-CKII) and knockdown plasmids (shCKII) were ordered from GeneChem (Shanghai, China). MK2206 and TBB were purchased from MedChemExpress (Monmouth Junction, NJ, USA). In general, the final concentrations of MK2206 and TBB were 25 µM and 10 µM respectively. The cells were treated with drugs for 24 h before proliferation-related tests or western blot analysis. The transfection reagent used for transient transfer was Lipofectamine 3000 (Invitrogen, Thermo Fisher Scientific, Inc., USA). For stable transfer, GeneChem (Shanghai, China) was used.

### Western blotting

Western blotting is a standard method to detect the total protein content of a sample. Here, the tissues or cells to be tested were lysed with RIPA buffer to generate a protein sample. Protein samples were separated on 10% SDS and transferred to polyvinylidene fluoride (PVDF) membranes. After the membranes were incubated in 5% skim milk for 1 h, the primary antibody was added for incubation overnight at 4 °C. Finally, the membranes were incubated with the secondary antibody for 1 h at room temperature and washed 2–3 times with TBST. Subsequently, a ChemiDoc XRS System (Bio–Rad) was used to capture images, and Quantity One software (Bio–Rad) was used to quantify the band densities. Primary antibodies against NCAPG (ab226805), PCNA (ab29), and CKII (ab 70,774) were purchased from Abcam (Cambridge, UK); those for AKT (9272S), p-AKT (9271S), PTEN (9552S), and p-PTEN (9551S) were purchased from Cell Signaling Technology; and the GAPDH antibody (60,004-1-lg) was purchased from Proteintech (Wuhan, China).

### Quantitative real-time polymerase chain reaction (qRT-PCR)

Quantitative real-time polymerase chain reaction was performed as described in a previous experiment [24]. The results of the analysis were the expression of each gene relative to the expression of a housekeeping gene. In in vitro studies, we used the 2^−Δ Δ CT^ method for calculations, and in tissues, we used 2^−ΔCT^ for the calculations. The primers used in the experiment were obtained from Shanghai Generay Biotech Co., Ltd. and are as follows: NCAPG, forward: 5ʹ-TCAAGGCTGGTTACGGTTCT-3ʹ, reverse: 5ʹ -GTCCCACCAGTTCACTGAGA-3ʹ; GAPDH, forward: 5ʹ-ACCCAGAAGACTGTGGATGG-3ʹ, reverse: 5ʹ-TCAGCTCAGGGATGACCTTG-3ʹ.

### Co-immunoprecipitation (CO-IP)

Co-IP was used to assess whether two proteins interacted. According to the manufacturer’s instructions, we used protein A/G PLUS-Agarose, purchased from Santa Cruz Biotechnology, to generate two sets of protein samples, the negative control group (IgG group) and the experimental group. The protein samples were separated by SDS–PAGE and tested by immunoblotting. The following antibodies were required: IgG (ab172730), purchased from Abcam (Cambridge, UK); and PTEN (22,034-1-AP), NCAPG (24,563-1-AP), and CKII (10,992-1-AP) from Proteintech (Wuhan, China).

### EdU incorporation assay

The 5-ethynyl-2ʹ-deoxyuridine (EdU) proliferation test is suitable to measure cell proliferation. We followed the manufacturer’s instructions and prepared a 50 µM EdU (RiboBio Biotechnology, Guangzhou, China) solution. One hundred microliters of this prepared liquid was added to each well of a plate, and the plate was incubated at room temperature for 2 h. Then, the cells were fixed with 4% polyoxymethylene for 30 min at room temperature. Subsequently, the cells were incubated with Apollo staining solution and Hoechst 33,342 for 30 min and then washed 2–3 times with PBS for 5 min each time. Finally, an Olympus fluorescence microscope (Tokyo, Japan) was used to observe five randomly selected fields of view and analyze the proliferation rate. After staining, the nuclei appear blue due to Hoechst 33,342, proliferating cells appear red from Apollo staining with EdU; the blue and red stained cells were superimposed. ImageJ was used to count the total number of cells and the number of proliferating cells.

### Cell Counting Kit-8 assay

A Cell Counting Kit-8 (CCK-8) assay was also used to measure cell proliferation. HCC cells (5000 cells/well) were seeded into a 96-well plate and cultured at 37 °C with 5% CO_2_. After culturing for 0, 24, 48, and 72 h, 10 µL of CCK-8 reagent (CCK-8; Dojindo Laboratories, Kumamoto, Japan) was added to each well followed by incubation at 37 °C with 5% CO_2_ for 2 h. A microplate reader (Bio–Rad, Berkeley, CA, USA) was used to measure the absorbance at 450 nm.

### Plate cloning

Plate cloning experiments were also used to measure cell proliferation. Hepatoma cells were seeded into a six-well plate at a density of 500 cells/well and cultured at 37 °C with 5% CO2. After 14 days, when cell aggregation was clearly visible to the naked eye, the cells were washed 2–3 times with PBS. Then, 1 mL of crystal violet staining solution was added to each well and stained for 1 h at room temperature. The staining solution was rinsed away before observation.

### Oncomine data analysis

Oncomine (http://www.oncomine.com) is the world's largest cancer gene chip database and integrated data mining platform, which aims to mine cancer gene information [25]. Through Oncomine, differential expression analysis and coexpression analysis can be carried out to identify differentially expressed genes in certain cancers, identify target genes and determine the direction of research. We compared the mRNA expression of NCAPG in liver cancer tissue and normal liver tissue in the liver cancer dataset. Three datasets were included in our study: Wurmbach et al. [26] and Roessler et al. (including the Roessler Liver 1 and Roessler Liver 2 datasets) [27]. We used an unpaired T test to analyze the difference in NCAPG expression between HCC and normal liver tissues. We assessed the relationship between NCAPG expression and overall survival according to the Guichard Liver dataset [28] by the log-rank (Mantel–Cox) test.

### Data analysis

All data were analyzed using SPSS 22.0. All experimental data are expressed as the mean ± standard deviation and were analyzed using Student’s t test and one-way analysis of variance. P < 0.05 indicates that there was a significant difference between groups.

## Results

### NCAPG is highly expressed in liver cancer tissues and is associated with poor prognosis

**Fig. 1:**
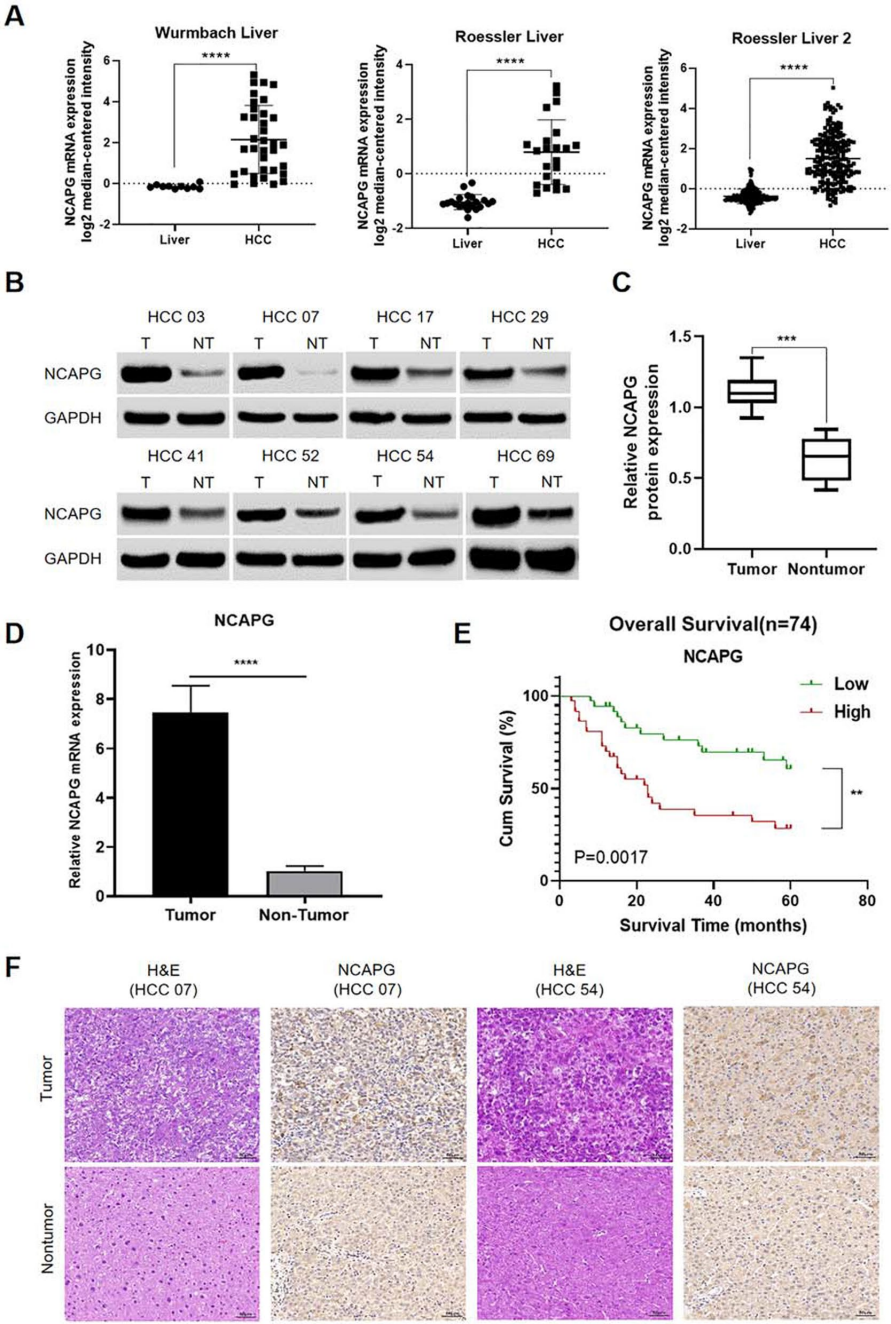
NCAPG is highly expressed in liver cancer tissues and is associated with poor prognosis. **A** The results from analysis of the three datasets selected from the Oncomine database (Dataset 1, Wurmbach Liver; Dataset 2, Roessler Liver 1; Dataset 3, Roessler Liver 2; ****P < 0.0001; independent Student’s t test) showed that NCAPG mRNA was overexpressed in HCC tumour tissues compared to adjacent nontumor tissues. **B** and **C** Western blot analysis of NCAPG in HCC tissues and adjacent nontumor tissues suggested that the protein levels of NCAPG were higher in tumour tissues. Quantitative analysis showed the same pattern (T, tumour; NT, nontumor). **D** NCAPG mRNA expression was examined in 85 pairs of primary liver cancer tissues and the corresponding normal adjacent tissues via qRT–PCR, which indicated that NCAPG mRNA was overexpressed in HCC tumour tissues compared to adjacent nontumor tissues. **E** Overall survival curves for two groups of patients with HCC: as NCAPG expression increased, patient prognosis worsened (Guichard Liver). **F** Immunohistochemical staining showed that NCAPG protein expression was higher in tumour tissue. Representative cases are shown (200 ×) (**P < 0.01, ***P < 0.001)

To evaluate the relationship between NCAPG and HCC, we first analysed three microarray datasets from the Oncomine database. The results showed that NCAPG was significantly overexpressed in most liver cancer tissues compared with the adjacent nontumor control tissues (Fig. 1A). TTo determine whether NCAPG is overexpressed in HCC, we examined NCAPG expression in 85 pairs of primary liver cancer tissues and the corresponding normal adjacent tissues. The Western blot results showed that compared with the adjacent nontumor tissues, the primary liver cancer tissues exhibited significant overexpression of NCAPG (Fig. 1B). The results obtained by qRT–PCR were consistent with these findings (Fig. 1C). In addition, IHC analysis revealed the same results; that is, NCAPG is highly expressed in liver cancer tissues (Fig. 1E).

To evaluate the relationship between NCAPG expression and the overall survival rate in patients with liver cancer, we analysed a microarray dataset from the Oncomine database, and the results showed that NCAPG expression was associated with a low overall survival rate (Fig. 1D), which was in line with our previous findings [29].

### NCAPG promotes the proliferation of liver cancer cells in vivo and in vitro

**Fig. 2:**
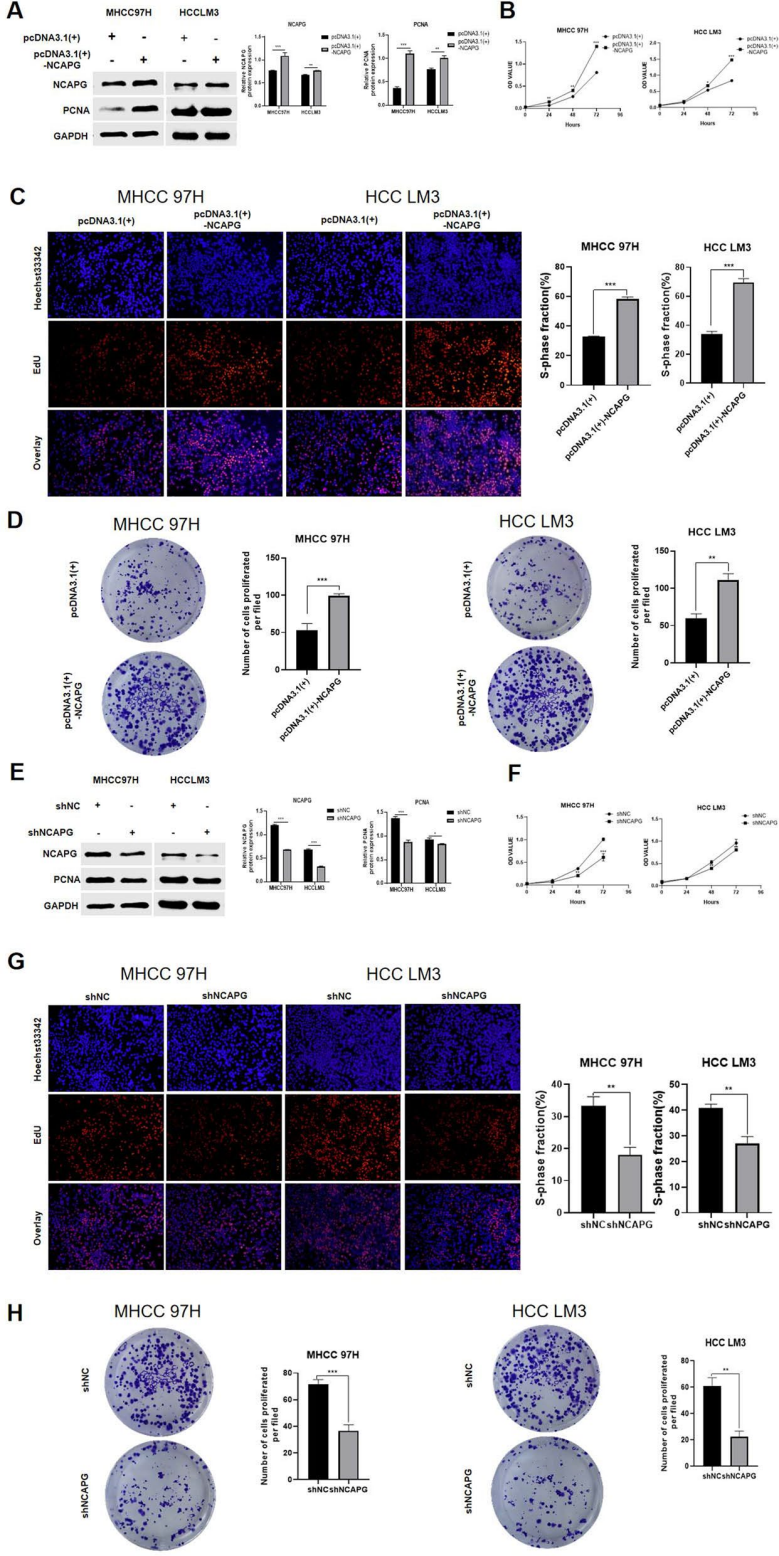
NCAPG promotes the proliferation of liver cancer cells in vitro. **A** NCAPG protein expression in HCC cells was detected by western blotting after transfection with the NCAPG overexpression plasmids (pcDNA3.1( +)-NCAPG) and pcDNA3.1( +). Quantitative analysis showed the same pattern. **B** CCK-8 assays showed that upregulation of NCAPG significantly increased the proliferation of MHCC97H and HCCLM3 cells (***P < 0.001). **C** and **D** EdU incorporation and plate colony formation assays showed that upregulation of NCAPG significantly increased the proliferation of MHCC97H and HCCLM3 cells (***P < 0.001). **E** Western blotting was used to detect the expression of NCAPG and PCNA in cells transfected with the NCAPG knockdown plasmid (shNCAPG) and shNC. Quantitative analysis showed the same pattern. **F**, **G** and **H** CCK-8, EdU incorporation and plate colony formation assays were used to detect the effects of NCAPG knockdown on cell proliferation, and NCAPG knockdown in cells was found to significantly decrease cell proliferation (*P < 0.05, **P < 0.01, ***P < 0.001; independent Student’s t test)

In previous studies, the expression of NCAPG in various liver cancer cells was screened. Therefore, MHCC97H and HCCLM3 cells were selected for this experiment due to their moderate expression of NCAPG [30]. To explore the role of NCAPG in the proliferation of liver cancer cells, we first stably transfected an NCAPG overexpression plasmid (pcDNA3.1( +)-NCAPG) into MHCC97H and HCCLM3 cells. The Western blot results showed that the expression of PCNA, a proliferation-related protein, in MHCC97H and HCCLM3 cells overexpressing NCAPG was higher than that in the corresponding control cells (Fig. 2A). Furthermore, we verified that the cell proliferation ability was significantly enhanced in the NCAPG overexpression group through EdU incorporation and CCK-8 assays (Fig. 2B and C). In addition, in the plate colony formation assay, we observed that cells with stable upregulation of NCAPG formed more cell clusters than control cells (Fig. 2D). Second, a NCAPG knockdown plasmid (shNCAPG) was transfected into MHCC97H and HCCLM3 cells, and the opposite effects were observed. In cells with NCAPG downregulation, the expression of PCNA was lower (Fig. 2E), the proliferation ability was weaker (Fig. 2F and G), and the number of cell clusters formed due to cell growth was lower (Fig. 2H).

**Fig. 3:**
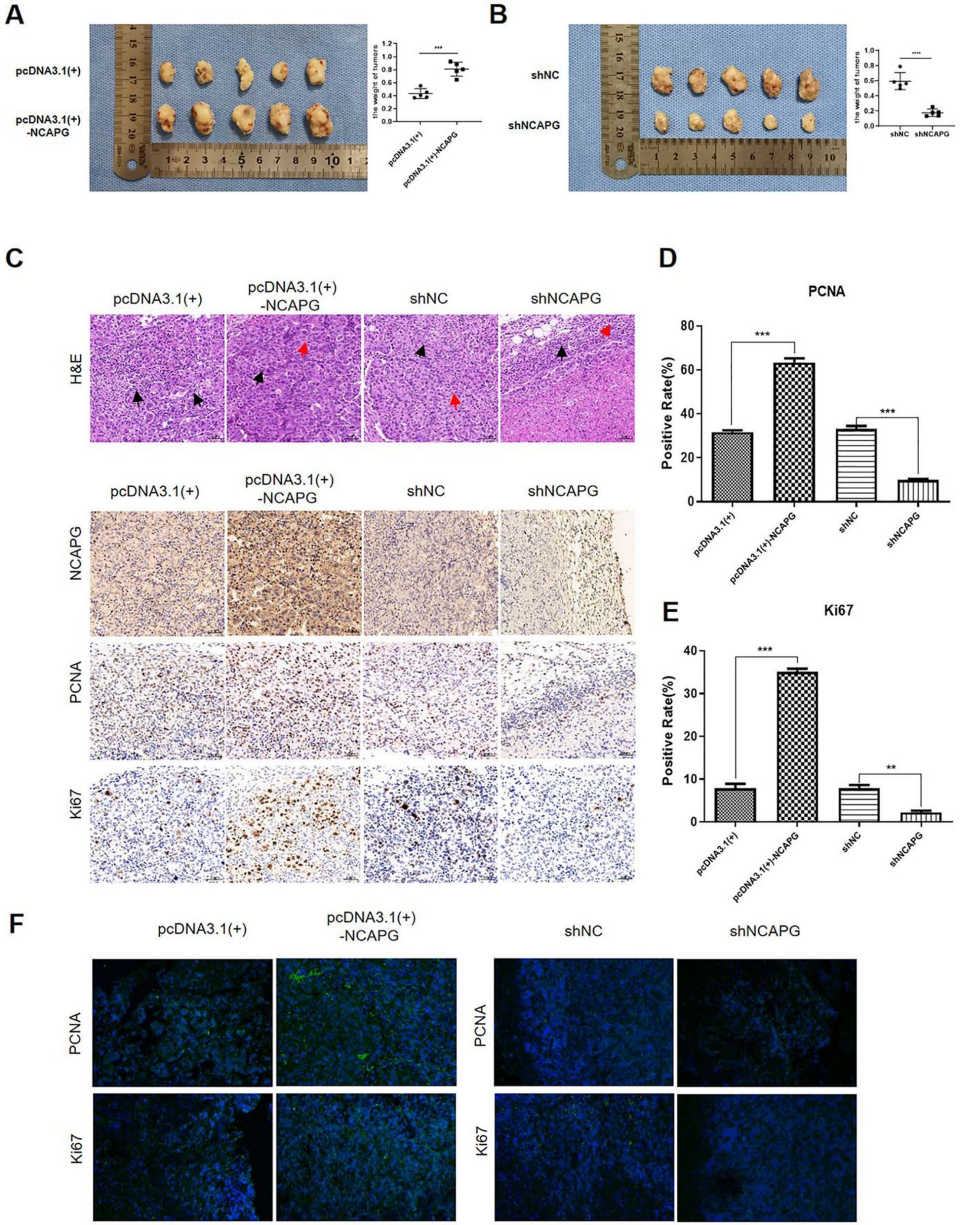
NCAPG promotes the proliferation of liver cancer cells in vivo. **A** The weights of tumours from mice injected with MHCC97H cells transfected with NCAPG overexpression and corresponding control plasmids (pcDNA3.1( +)-NCAPG) and pcDNA3.1( +), respectively) showed a positive correlation with the expression of NCAPG. **B** The weights of tumours from mice injected with MHCC97H cells transfected with the NCAPG knockdown plasmid (shNCAPG) and shNC showed a positive correlation with the expression of NCAPG. **C**, **D** and **E** Immunohistochemical analysis and HE staining of tissues from mice in the NCAPG overexpression group and NCAPG knockdown group showing that NCAPG can promote tumour proliferation. Quantitative analysis showed the same result. **F** The FISH assay of samples from mice in the NCAPG overexpression group and NCAPG knockdown group showed that tumour proliferation was positively correlated with the expression of NCAPG (**P < 0.01, ***P < 0.001)

Then, we studied the effects of modulating the expression of NCAPG on cell proliferation in vivo. Twenty mice were randomly divided into 4 groups (NCAPG overexpression and control; NCAPG knockdown and control), with 5 mice in each group. The results showed that the average tumour volumes in mice injected with cells overexpressing NCAPG were significantly greater than those in control mice (Fig. 3A). To further confirm the effects of NCAPG on the proliferation of liver cancer cells in vivo, we removed the tumours for HE staining and IHC analysis. The results showed that the masses consisted of only tumour tissue and that the tumours that were removed from mice injected with NCAPG-overexpressing cells had higher expression levels of NCAPG, PCNA, and Ki-67 than those removed from mice in the control group (Fig. 3C). Comparing the group with NCAPG downregulation and its corresponding control, the results were reversed. In mice injected with NCAPG knockdown cells, the tumours weighed less (Fig. 3B) and the expression levels of NCAPG, PCNA, and Ki-67 were lower than those in the corresponding control mice (Fig. 3C, D and E). Moreover, the FISH assay produced the same results (Fig. 3F). In summary, the above data indicated that NCAPG can promote the proliferation of liver cancer cells in vivo.

### Proteomics analysis and transcriptome sequencing in this study

We identified that NCAPG has an important role in the proliferation of HCC. However, its mechanism of action has not yet been elucidated. To determine the mechanisms involved, we performed proteomic analysis and transcriptome sequencing.

**Fig. 4:**
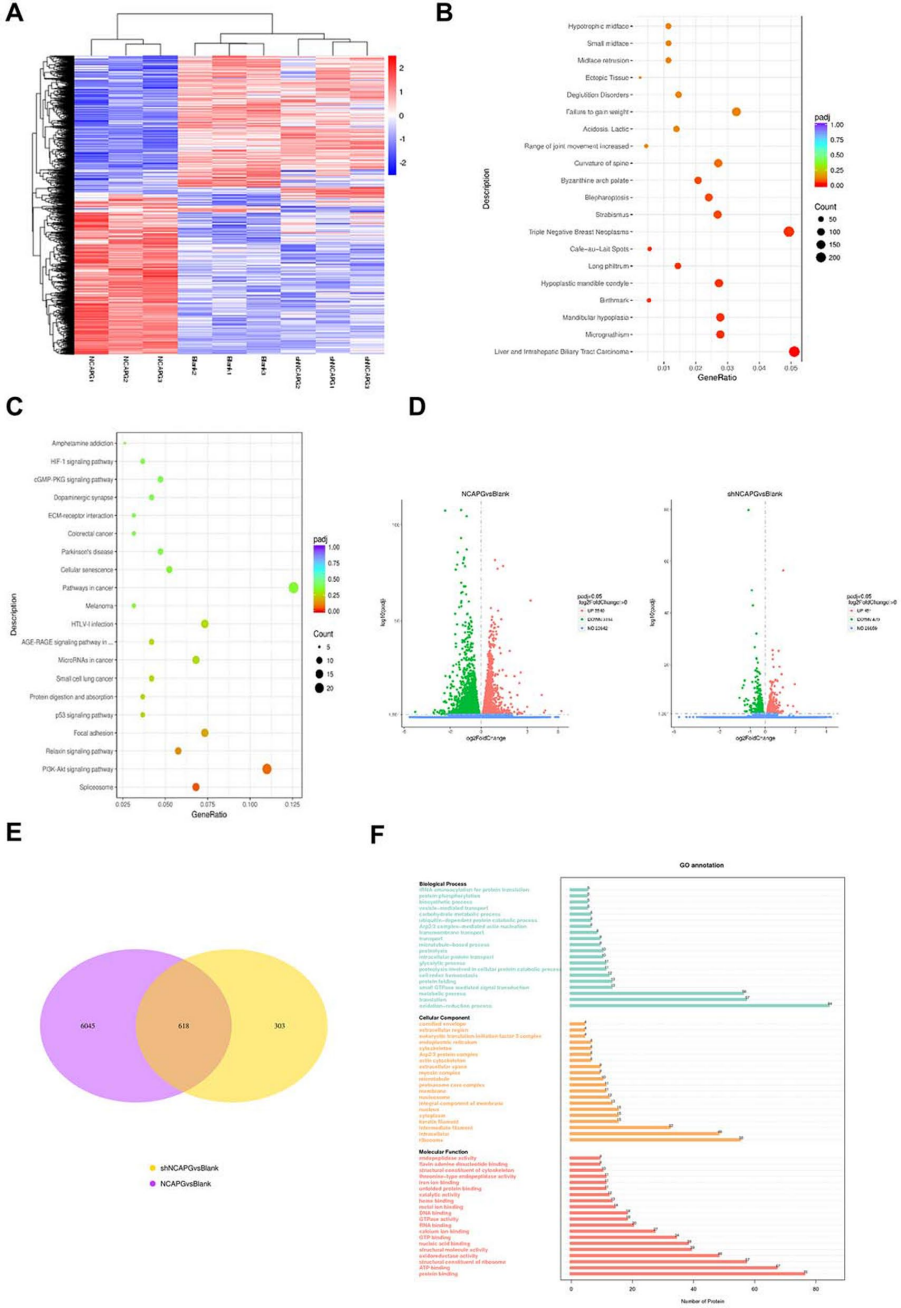
Proteomic analysis and transcriptome sequencing. **A** Cluster analysis of differentially expressed genes indicated that the expression of multiple genes changed with up/downregulation of NCAPG (red indicates high gene expression, blue indicates low gene expression). **B** Enrichment analysis with DisGeNET suggested that abnormal expression of NCAPG was indeed closely correlated with liver-related tumor diseases (the size of the dot represents the number of genes annotated to the DisGeNET term, and the color from red to purple represents the significance of enrichment). **C** The KEGG enrichment analysis results indicated that there was a significant correlation between the NCAPG gene and the PI3K-AKT pathway (the size of each dot in the figure represents the number of genes annotated to the KEGG pathway, and the colour from red to purple represents increasing significance of enrichment). **D** Volcano plot showing that 3549 genes were upregulated and 3114 genes were downregulated when NCAPG was upregulated and that 451 genes were upregulated and 470 genes were downregulated when NCAPG was knocked down. **E** Venn diagram showing 618 differentially expressed genes. **F** Proteomic analysis indicated that CKII was one of five genes involved in the participation of NCAPG in protein phosphorylation

Enrichment analysis with DisGeNET showed that the role of NCAPG in liver cancer is more important than that in other diseases (Fig. 4B). This result was consistent with the experimental results mentioned above. The transcriptome sequencing results revealed that 3549 genes were upregulated and 3114 genes were downregulated when NCAPG was upregulated (Fig. 4D). When NCAPG was downregulated, 451 genes were upregulated, and 470 genes were downregulated (Fig. 4D). In summary, there were changes in the expression levels of 6663 genes when NCAPG was upregulated and of 921 genes when NCAPG was downregulated (Fig. 4A). Among the differentially expressed genes, the expression of 618 changed regardless of whether NCAPG was upregulated or downregulated (Fig. 4E). The results of the differential gene expression analysis of liver cancer cells with NCAPG downregulation indicated that the expression of PTEN was significantly increased compared with that in the control cells (P < 0.001). In addition, the KEGG pathway enrichment analysis results suggested that there was a significant correlation between NCAPG and the PI3K-AKT pathway (Fig. 4C).

From the transcriptome sequencing results described above, it can be inferred that NCAPG has an impact on PTEN transcription. To reveal the in-depth relationship between NCAPG and PTEN, we conducted proteomic analysis on proteins that could interact with NCAPG. We identified hundreds of proteins that can interact with NCAPG. Among these proteins, CKII was most closely related to the PI3K-AKT signalling pathway and PTEN (Fig. 4F). Therefore, we hypothesized that NCAPG may affect the transcription and expression of PTEN through CKII.

### NCAPG enhances the proliferation of HCC and alters the PI3K-AKT pathway by affecting PTEN expression

**Fig. 5:**
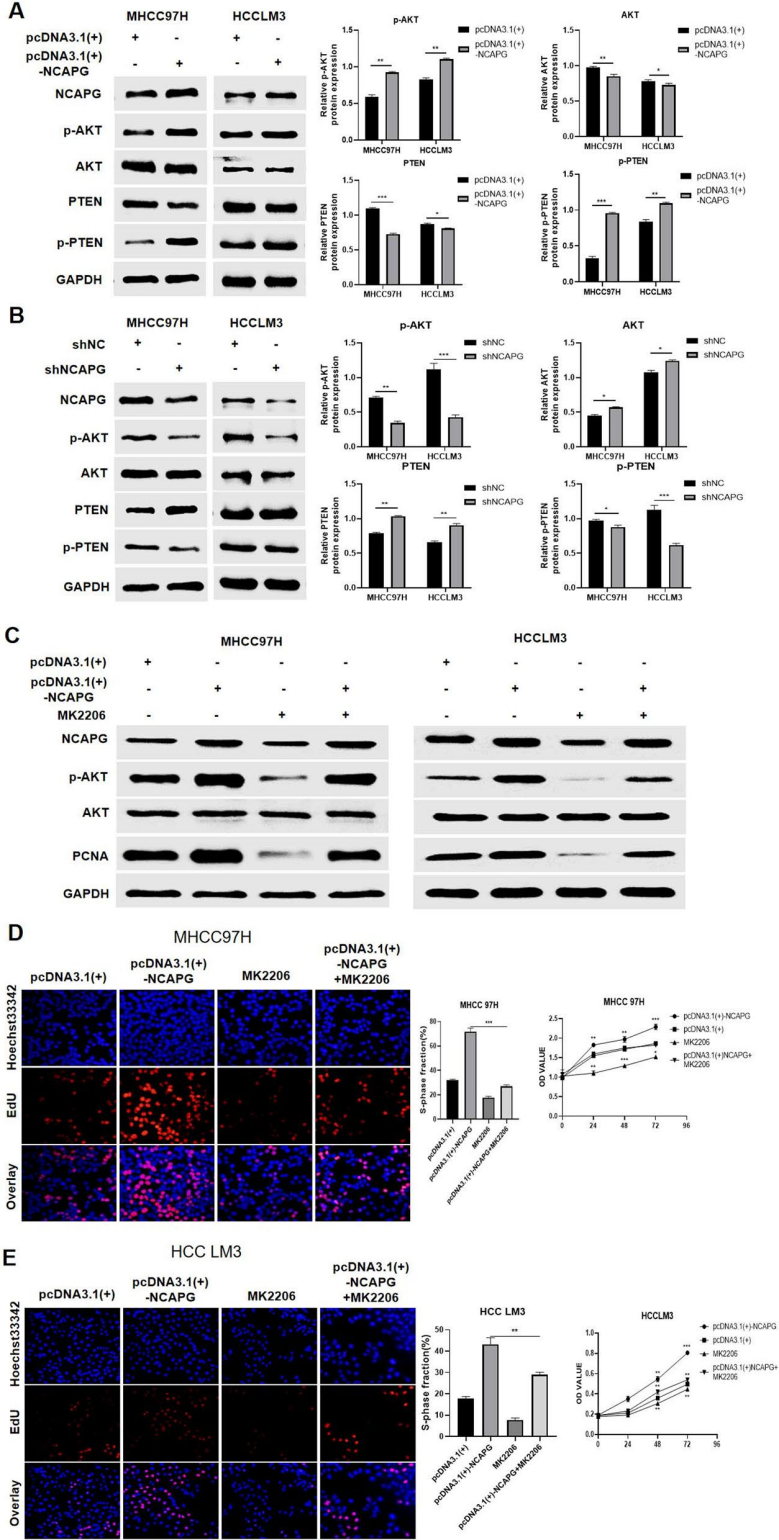
NCAPG enhances the proliferation of HCC cells and affects the PI3K-AKT pathway by affecting the expression of PTEN. **A** Western blot analysis showing the levels of PTEN, AKT, p-PTEN and p-AKT when NCAPG was overexpressed. Quantitative analysis showed the same pattern. **B** Western blot analysis showing the levels of PTEN, AKT, p-PTEN and p-AKT when NCAPG was knocked down. Quantitative analysis showed the same pattern. **C** When MK2206 was added to NCAPG-overexpressing cells, the levels of AKT and p-AKT were reversed. **D** and **E** The cell proliferation ability of HCCLM3 and MHCC97H cells, as evaluated by EdU incorporation and CCK-8 assays, was attenuated when NCAPG was upregulated with MK2206. The values indicate the means ± SDs of three independent experiments (*P < 0.05, **P < 0.01, ***P < 0.001; independent Student’s t test)

Our transcriptome sequencing results indicated that the PI3K-AKT pathway plays an important role in the mechanism by which NCAPG enhances the proliferation of hepatocellular carcinoma (Fig. 4C). The transcriptome sequencing results showed that NCAPG expression was negatively correlated with PTEN expression and that NCAPG promoted the phosphorylation of proteins in the PI3K-AKT pathway. To clarify the analysis results, Western blotting was used to measure the levels of AKT, p-AKT, PTEN and p-PTEN in cells with NCAPG upregulation. The results showed that in cells overexpressing NCAPG, the levels of p-AKT and p-PTEN were incereased, while PTEN and AKT were expressed at low levels (Fig. 5A). In contrast, in NCAPG-silenced cells, the levels of p-AKT and p-PTEN were decreased, while those of PTEN and AKT were increased (Fig. 5B).

To reveal whether NCAPG affects the proliferation of HCC through PTEN and the PI3K-AKT pathway, we investigated whether the PI3K-AKT pathway inhibitor MK2206 could attenuate the proliferation of liver cancer. Cells transfected with pcDNA3.1( +)-NCAPG and treated with 25 μM MK2206 for 24 h were subjected to Western blot analysis. Interestingly, the Western blot results showed that MK2206 reduced the levels of p-AKT and p-PTEN in NCAPG-overexpressing cells (Fig. 5C). In addition, the EdU incorporation and CCK-8 assays showed that MK2206 attenuated cell proliferation induced by NCAPG overexpression (Fig. 5D and E). In summary, these data indicated that the effects of NCAPG on the proliferation of liver cancer cells are achieved through the PI3K-AKT signalling pathway and are closely related to PTEN.

### NCAPG enhances the phosphorylation of PTEN through CKII

**Fig. 6:**
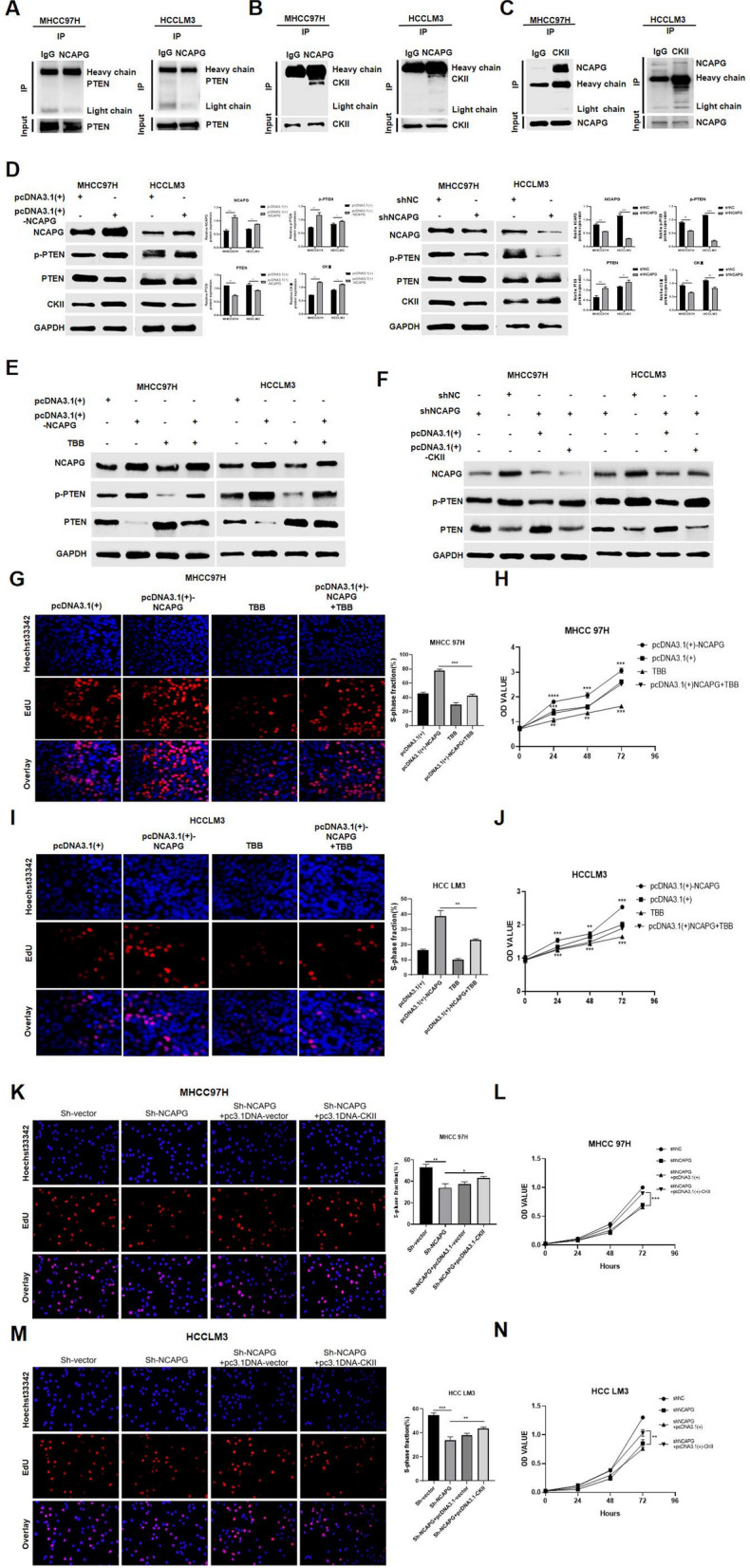
NCAPG enhances the phosphorylation of PTEN through CKII. **A** A Co-IP assay showed that endogenous NCAPG and PTEN did not bind directly. **B** Co-IP assay of endogenous NCAPG and CKII in MHCC97H and HCCLM3 cells. CKII was detected in the immunoprecipitate when the anti-NCAPG antibody was used as bait. **C** NCAPG was detected in the immunoprecipitate when the anti-CKII antibody was used as bait. **D** Western blot analysis showing the levels of PTEN, p-PTEN and CKII when NCAPG was upregulated or knocked down. Quantitative analysis showed the same pattern. **E** TBB was added to NCAPG-overexpressing cells, and Western blot analysis showed that the levels of PTEN, p-PTEN and CKII were reversed. **F** Transfection with the CKII overexpression plasmid combined with NCAPG silencing. Western blotting was used to measure the levels of PTEN, p-PTEN and CKII, and it was found that the levels of PTEN, p-PTEN and CKII were reversed. **G** to **J** The proliferation ability of HCCLM3 and MHCC97H cells was reversed when TBB was added to NCAPG-overexpressing cells, as determined by EdU incorporation and CCK-8 assays. The values indicate the mean ± SD of three independent experiments (*P < 0.05, **P < 0.01, ***P < 0.001; independent Student’s t test). **K** to **N** The proliferation ability of HCCLM3 and MHCC97H cells, as determined by EdU incorporation and CCK-8 assays, was reversed when NCAPG was silenced and CKII was upregulated. The values indicate the mean ± SD of three independent experiments (*P < 0.05, **P < 0.01, ***P < 0.001; independent Student’s t test)

We first found through Co-IP analysis that NCAPG and PTEN do not bind directly (Fig. 6A); that is, NCAPG cannot directly affect the transcription or phosphorylation of PTEN. Subsequently, we identified the key protein CKII from the proteomic analysis results and speculated that CKII is involved in the effects of NCAPG on PTEN (Fig. 4F).

**Fig. 7:**
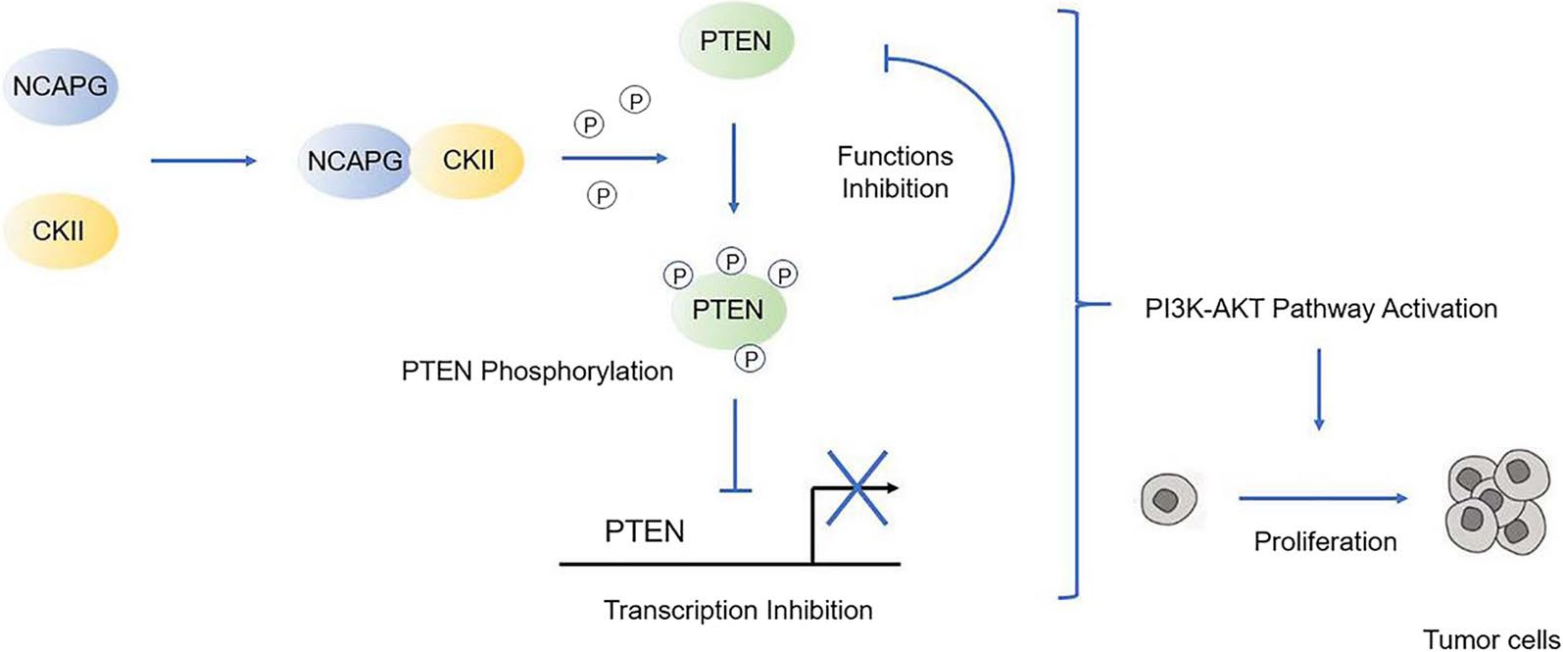
A schematic model showing the mechanism by which NCAPG promotes the proliferation of HCC cells. NCAPG can promote the phosphorylation of PTEN through interaction with CKII, because the interaction between the NCAPG and CKII proteins has been proven. This phenomenon can inhibit the transcription and functions of PTEN and activate the PI3-AKT signalling pathway, ultimately leading to the promotion of HCC proliferation

Then, we confirmed the interaction between the NCAPG and CKII proteins by Co-IP (Fig. 6B and C). Cells overexpressing NCAPG and cells with silenced NCAPG were analysed by Western blotting. The results showed that the levels of CKII and p-PTEN were high in cells overexpressing NCAPG (Fig. 6D). Conversely, the levels of CKII and p-PTEN in cells with silenced NCAPG were reduced (Fig. 6D). To further confirm the key role of CKII, we used the CKII inhibitor TBB and cells overexpressing CKII to determine whether CKII plays an important role in the effects of NCAPG on PTEN. Proliferation assays and Western blot analysis were carried out in NCAPG-overexpressing cells after TBB treatment. In addition, the CKII overexpression plasmid was transfected into NCAPG-silenced cells for subsequent proliferation assays and Western blot analysis. The Western blot results showed that when the expression of CKII was inhibited by TBB, the effect of NCAPG overexpression to promote PTEN phosphorylation was reversed (Fig. 6E), while CKII overexpression had the opposite effect in NCAPG-silenced cells (Fig. 6F). In addition, inhibition of CKII expression weakened the increase in the proliferation ability of HCC cells induced by NCAPG overexpression (Fig. 6G–J). In contrast, CKII overexpression reversed the decrease in cell proliferation caused by NCAPG silencing (Figs. 6K–N). These experimental data showed that CKII is indeed the key component linking NCAPG and PTEN (Fig. 7).

## Discussion

Cell proliferation is the basis of cell growth and development as well as heredity and reproduction. Cell proliferation is not unlimited, as it is controlled by a regulatory mechanism in the human body. Once this regulation is lost, uncontrollable cell proliferation occurs. Malignant tumour cells proliferate endlessly, causing loss of cell morphology and function and resulting in local infiltration, distant metastasis, etc., which affect human health. Therefore, understanding the mechanism of HCC proliferation will help to develop newer and better treatment strategies to improve the survival rate of patients with HCC.

We found that NCAPG can enhance the proliferation of HCC cells. Studies have shown that NCAPG is involved in the pathogenesis of a variety of cancers, including prostate cancer [6], paediatric glioma [30], renal cell carcinoma [31], multiple myeloma [32] and melanoma [33]. Through bioinformatics analysis, it was found that NCAPG is also a key gene involved in the occurrence and development of liver cancer. In this study, we further confirmed differences in NCAPG expression in 85 pairs of primary HCC tissues and the corresponding adjacent normal tissues through IHC and Western blot analyses. These data suggest that NCAPG may be a key oncogene. In addition, our experimental data showed that NCAPG overexpression promotes the proliferation of liver cancer cells, while NCAPG silencing inhibits the proliferation of liver cancer cells. Moreover, there were significant differences in tumour volumes in the nude mouse subcutaneous tumorigenesis assay. Thus, we can confidently assume that NCAPG plays an important role in tumour proliferation.

The mechanism by which NCAPG enhances the proliferation of HCC cells—that is, CKII-dependent regulation of PTEN—has been proven. PTEN is currently recognized as an extremely important tumour suppressor gene, and it has been confirmed that abnormal regulation of PTEN is an important link in tumorigenesis and tumour development [11]. In this study, for the first time, we found that NCAPG is the upstream gene affecting the expression of PTEN and confirmed the regulation of PTEN by NCAPG. NCAPG not only inhibits PTEN expression through interaction with CKII but also interferes with its function through PTEN phosphorylation. This negative feedback regulation significantly inhibits the function and expression of PTEN to activate the PI3K-AKT pathway. At present, many studies are exploring antitumor strategies based on PTEN, such as the inhibitory effect of TSPAN7 on bladder cancer [34]. These studies are also based on the regulatory effect of a specific gene on PTEN. Therefore, based on our findings, we believe that the regulation of PTEN by NCAPG may also have the potential to become a direction in antitumor research.

CKII is a phosphorylated kinase that is involved in many processes related to tumorigenesis and tumour development [35–38]. Our study is the first to reveal and confirm the protein–protein interaction between NCAPG and CKII, which has not been reported previously. By regulating CKII, NCAPG interferes with a series of downstream molecular processes, such as the expression and phosphorylation of PTEN, and regulates the PI3K-AKT pathway.

In this study, the correlations among NCAPG, PTEN and CKII were revealed by transcriptome sequencing and proteomic analysis. Both of these methods can help researchers reveal interactions between genes and proteins. Through transcriptome sequencing, we found that NCAPG affects the proliferation of liver cancer cells, while proteomic analysis helped us to find that NCAPG regulates the key PTEN protein CKII. These complementary methods based on big data improve the accuracy of the study design and can help us to better show the involvement of NCAPG in the promotion of liver cancer.

We believe that NCAPG-targeted therapies might be feasible, because the molecular mechanisms driving the tumorigenic effects of NCAPG have been a focus. In our previous study, we found that microRNA-181c can suppress the growth and metastasis of hepatocellular carcinoma by modulating NCAPG expression [39]. This was a promising result showing that small molecule drugs targeting NCAPG are potentially feasible. Therefore, the identification of genes such as NCAPG might be performed in the future, and these results could help us to select small molecule drugs to improve HCC patient outcomes. However, both challenges and opportunities are present. The greatest challenge for NCAPG-targeted therapies has been the indefinite R&D cycle. The development of small molecule drugs targeting NCAPG may not be rapid. Therefore, more research is needed at this time.

In conclusion, for the first time, our research revealed the molecular mechanism by which NCAPG promotes liver cancer proliferation, namely, NCAPG inhibits PTEN through interaction with CKII and then activates the PI3K-AKT pathway to promote the proliferation of liver cancer. Our study further reveals the molecular biological role of NCAPG as an oncogene and provides a basis for subsequent research.

## Data Availability

The datasets used and/or analysed during the current study are available from the corresponding author on reasonable request.

## Abbreviations

HCC: Hepatocellular carcinoma
NCAPG: Non-SMC Subunit in the Concentrate I Complex
PTEN: Phosphatase and Tensin Homolog
CKII: Casein Kinase 2 Alpha 1
FISH: Fluorescence in situ hybridization
EdU: 5-Ethynyl-2ʹ-deoxyuridine
CO-IP: Co-immunoprecipitation
CCK-8: Cell Counting Kit-8

## References

[1] Sung H, Ferlay J, Siegel RL, Laversanne M, Soerjomataram I, Jemal A, Bray F (2021). Global cancer statistics 2020: GLOBOCAN estimates of incidence and mortality worldwide for 36 cancers in 185 countries. CA-A Cancer J Clin.

[2] Bruix J, Gores GJ, Mazzaferro V (2014). Hepatocellular carcinoma: clinical frontiers and perspectives. Gut.

[3] Sia D, Villanueva A, Friedman SL, Llovet JM (2017). Liver cancer cell of origin, molecular class, and effects on patient prognosis. Gastroenterology.

[4] Eberlein A, Takasuga A, Setoguchi K, Pfuhl R, Flisikowski K, Fries R, Klopp N, Fuerbass R, Weikard R, Kuehn C (2009). Dissection of genetic factors modulating fetal growth in cattle indicates a substantial role of the non-SMC Condensin I Complex, Subunit G (NCAPG) Gene. Genetics.

[5] Yan H, Li Z, Shen Q, Wang Q, Tian J, Jiang Q, Gao L (2017). Aberrant expression of cell cycle and material metabolism related genes contributes to hepatocellular carcinoma occurrence. Pathol Res Pract.

[6] Goto Y, Kurozumi A, Arai T, Nohata N, Kojima S, Okato A, Kato M, Yamazaki K, Ishida Y, Naya Y, Ichikawa T, Seki N (2017). Impact of novel miR-145-3p regulatory networks on survival in patients with castration-resistant prostate cancer. Br J Cancer.

[7] Wang Y, Gao B, Tan PY, Handoko YA, Sekar K, Deivasigamani A, Seshachalam VP, OuYang HY, Shi M, Xie C, Goh BKP, Ooi LL, Hui KM (2017). A genome-wide CRISPR cell growth screen identifies NCAPG as a new therapeutic target for hepatocellular carcinoma. Ann Oncol.

[8] Gong C, Ai J, Fan Y, Gao J, Liu W, Feng Q, Liao W, Wu L (2019). NCAPG promotes the proliferation of hepatocellular carcinoma through PI3K/AKT signaling. Onco Targets Ther.

[9] Hollander MC, Blumenthal GM, Dennis PA (2011). PTEN loss in the continuum of common cancers, rare syndromes and mouse models. Nat Rev Cancer.

[10] Lee Y-R, Chen M, Pandolfi PP (2018). The functions and regulation of the PTEN tumour suppressor: new modes and prospects. Nat Rev Mol Cell Biol.

[11] Chalhoub N, Baker SJ (2009). PTEN and the PI3-kinase pathway in cancer. Annu Rev Pathol-Mech Dis.

[12] Gu J, Ou W, Huang L, Wu J, Li S, Xu J, Feng J, Liu B, Zhou Y (2016). PTEN expression is associated with the outcome of lung cancer: evidence from a meta-analysis. Minerva Med.

[13] Salmena L (2016). PTEN: history of a tumor suppressor. Methods Mol Biol.

[14] Pinna LA (2002). Protein kinase CK2: a challenge to canons. J Cell Sci.

[15] Litchfield DW (2003). Protein kinase CK2: structure, regulation and role in cellular decisions of life and death. Biochemical J.

[16] Leslie NR, Batty IH, Maccario H, Davidson L, Downes CP (2008). Understanding PTEN regulation: PIP2, polarity and protein stability. Oncogene.

[17] Morotti A, Panuzzo C, Crivellaro S, Carra G, Fava C, Guerrasio A, Pandolfi PP, Saglio G (2015). BCR-ABL inactivates cytosolic PTEN through Casein Kinase II mediated tail phosphorylation. Cell Cycle.

[18] Chen L, Liu S, Tao Y (2020). Regulating tumor suppressor genes: post-translational modifications. Signal Transduct Target Ther.

[19] Bolduc D, Rahdar M, Tu-Sekine B, Sivakumaren SC, Raben D, Amzel LM, Devreotes P, Gabelli SB, Cole P (2013). Phosphorylation-mediated PTEN conformational closure and deactivation revealed with protein semisynthesis. eLife.

[20] Torres J, Pulido R (2001). The tumor suppressor PTEN is phosphorylated by the protein kinase CK2 at its C terminus—Implications for PTEN stability to proteasome-mediated degradation. J Biol Chem.

[21] Puckett MC, Goldman EH, Cockrell LM, Huang B, Kasinski AL, Du Y, Wang C-Y, Lin A, Ichijo H, Khuri F, Fu H (2013). Integration of apoptosis signal-regulating kinase 1-mediated stress signaling with the Akt/Protein Kinase B-I kappa B Kinase cascade. Mol Cell Biol.

[22] Ghosh-Choudhury N, Mandal CC, Ghosh-Choudhury N, Choudhury GG (2010). Simvastatin induces derepression of PTEN expression via NF kappa B to inhibit breast cancer cell growth. Cell Signal.

[23] Akgun S, Kucuksayan H, Ozes ON, Can O, Alikanoglu AS, Yildiz M, Akca H (2019). NF-kappa B-induced upregulation of miR-548as-3p increases invasion of NSCLC by targeting PTEN. Anticancer Agents Med Chem.

[24] Min J, Feng Q, Liao W, Liang Y, Gong C, Li E, He W, Yuan R, Wu L (2018). IFITM3 promotes hepatocellular carcinoma invasion and metastasis by regulating MMP9 through p38/MAPK signaling. FEBS Open Bio.

[25] Rhodes DR, Yu JJ, Shanker K, Deshpande N, Varambally R, Ghosh D, Barrette T, Pandey A, Chinnaiyan AM (2004). ONCOMINE: a cancer microarray database and integrated data-mining platform. Neoplasia.

[26] Wurmbach E, Chen Y-B, Khitrov G, Zhang W, Roayaie S, Schwartz M, Fiel I, Thung S, Mazzaferro V, Bruix J, Bottinger E, Friedman S, Waxman S, Llovet JM (2007). Genome-wide molecular profiles of HCV-induced dysplasia and hepatocellular carcinoma. Hepatology.

[27] Roessler S, Jia H-L, Budhu A, Forgues M, Ye Q-H, Lee J-S, Thorgeirsson SS, Sun Z, Tang Z-Y, Qin L-X, Wang XW (2010). A unique metastasis gene signature enables prediction of tumor relapse in early-stage hepatocellular carcinoma patients. Can Res.

[28] Guichard C, Amaddeo G, Imbeaud S, Ladeiro Y, Pelletier L, Ben Maad I, Calderaro J, Bioulac-Sage P, Letexier M, Degos F, Clement B, Balabaud C, Chevet E, Laurent A, Couchy G, Letouze E, Calvo F, Zucman-Rossi J (2012). Integrated analysis of somatic mutations and focal copy-number changes identifies key genes and pathways in hepatocellular carcinoma. Nat Genet.

[29] Liu W, Liang B, Liu H, Huang Y, Yin X, Zhou F, Yu X, Feng Q, Li E, Zou Z, Wu L (2017). Overexpression of non-SMC condensin I complex subunit G serves as a promising prognostic marker and therapeutic target for hepatocellular carcinoma. Int J Mol Med.

[30] Liang M-L, Hsieh T-H, Ng K-H, Tsai Y-N, Tsai C-F, Chao M-E, Liu D-J, Chu S-S, Chen W, Liu Y-R, Liu R-S, Lin S-C, Ho DM-T, Wong T-T, Yang M-H, Wang H-W (2016). Downregulation of miR-137 and miR-6500-3p promotes cell proliferation in pediatric high-grade gliomas. Oncotarget.

[31] Yamada Y, Arai T, Kojima S, Sugawara S, Kato M, Okato A, Yamazaki K, Naya Y, Ichikawa T, Seki N (2018). Regulation of antitumor miR-144-5p targets oncogenes: Direct regulation of syndecan-3 and its clinical significance. Cancer Sci.

[32] Cohen Y, Gutwein O, Garach-Jehoshua O, Bar-Haim A, Kornberg A (2014). The proliferation arrest of primary tumor cells out-of-niche is associated with widespread downregulation of mitotic and transcriptional genes. Hematology.

[33] Ryu B, Kim DS, DeLuca AM, Alani RM (2007). Comprehensive expression profiling of tumor cell lines identifies molecular signatures of melanoma progression. PLoS ONE.

[34] Yu X, Li S, Pang M, Du Y, Xu T, Bai T, Yang K, Hu J, Zhu S, Wang L, Liu X (2021). TSPAN7 exerts anti-tumor effects in bladder cancer through the PTEN/PI3K/AKT pathway. Front Oncol.

[35] Lou DY, Dominguez I, Toselli P, Landesman-Bollag E, O'Brien C, Seldin DC (2008). The alpha catalytic subunit of protein kinase CKZ is required for mouse embryonic development. Mol Cell Biol.

[36] Meggio F, Pinna LA (2003). One-thousand-and-one substrates of protein kinase CK2?. FASEB J.

[37] St-Denis NA, Litchfield DW (2009). From birth to death: the role of protein kinase CK2 in the regulation of cell proliferation and survival. Cell Mol Life Sci.

[38] Borgo C, Ruzzene M (2019). Role of protein kinase CK2 in antitumor drug resistance. J Exp Clin Cancer Res.

[39] Jiyuan A, Gong C, Junjun W, Gao J, Liu W, Liao W, Linquan W (2019). MicroRNA 181c suppresses growth and metastasis of hepatocellular carcinoma by modulating NCAPG. Cancer Manag Res..

